# Personal

**Published:** 1915-02

**Authors:** 


### PERSONAL.

---

Announcement of removal, travel, and other matters of interest are requested. Please report errors in the listing of any physician in the State and other directories, that we may co-operate with the State Society in securing a correct list.

---

Drs. Reeve B. Howland and Charles Haase were appointed members of the Board of Health of Elmira on January 1, 1915.

---

Dr. Clyde Carey entertained the Elmira Clinic Society on January 18th. Subject of his paper was "Hypermetropia."

---

Dr. Anna Stuart has been appointed Bacteriologist of Elmira for 1915.

Dr. Eugene N. Ringueberg of Lockport announces the removal of his office to 13-15 Main Street, over Bayliss & Sweet's drug store.

---

Dr. E. T. Wentworth announces his location at 1232 Lake Avenue, Rochester.

---

Dr. Wm. Preiss of Buffalo announces that, on May 1, his office and residence will be removed to 304 W. Utica street.

---

Dr. Mary Innis Denton, formerly of Buffalo, now of Oxford University, Ohio, visited in Buffalo in December.

---

Dr. George W. Crile of Cleveland, is expected to join the Red Cross movement in Paris, to institute general measures to reduce the mortality from shock.

---

Dr. Theodore V. Bauer had a new automobile stolen on the night of January 19, and recovered the next day.

---

Dr. C. B. Vogt of Brooklyn spent the holidays in Niagara Falls.

---

Dr. C. A. Wisch of Niagara Falls visited the Mayo clinics in Rochester, Minn., in January.

---

Dr. Warren E. Esslie of Cleveland spent two weeks in Niagara Falls, about the beginning of the year.

---

Dr. Walter A. Scott of Niagara Falls has returned from a course of study in X-Ray work at the Post Graduate Hospital of New York.

---

As we go to press with this section, we learn with deep regret, of the very serious injury to Dr. Warren Britt of Tonawanda. Late in the afternoon of January 25, his automobile was struck by a Niagara Falls trolley, in Tonawanda. He sustained a fracture of four ribs and a concussion of the brain.

